# Plant-feeding may explain why the generalist predator *Euseius stipulatus* does better on less defended citrus plants but *Tetranychus*-specialists *Neoseiulus californicus* and *Phytoseiulus persimilis* do not

**DOI:** 10.1007/s10493-020-00588-x

**Published:** 2021-01-22

**Authors:** Joaquín Cruz-Miralles, Marc Cabedo-López, Michela Guzzo, Victoria Ibáñez-Gual, Víctor Flors, Josep A. Jaques

**Affiliations:** 1 Departament de Ciències Agràries i del Medi Natural, Universitat Jaume I (UJI), Castelló de la Plana, Spain; 2 Departament de Matemàtiques, Universitat Jaume I, UJI, Campus del Riu Sec, 12071 Castelló de la Plana, Spain; 3 Integración Metabólica y Señalización Celular, Departament de Ciències Agràries i del Medi Natural, Universitat Jaume I (UJI), Castelló de la Plana, Spain

**Keywords:** Phytoseiidae, Zoophytophagy, Plant defense, Sour orange, Cleopatra mandarin

## Abstract

The generalist predator *Euseius stipulatus* (Athias-Henriot) and the Tetranychidae-specialist predators *Neoseiulus californicus* (McGregor) and *Phytoseiulus persimilis* Athias-Henriot play a key role in the regulation of *Tetranychus urticae* Koch in Spanish citrus orchards. Previous studies have shown that sour orange (*Citrus aurantium* L.) and Cleopatra mandarin (*Citrus reshni* hort. ex Tan.) display extreme resistance and susceptibility to *T. urticae*, respectively. When offered a choice between these two genotypes infested by *T. urticae*, *E. stipulatus* preferred Cleopatra mandarin, whereas the specialists did not show any preference. The present study was undertaken to check whether these preferences could be related to the feeding of *E. stipulatus* on the host plant and/or to differences in prey feeding on the two plants. Our results demonstrate that *E. stipulatus* is a zoophytophagous mite, which can engage in direct plant feeding in sour orange and Cleopatra mandarin, whereas neither *N. californicus* nor *P. persimilis* do so. Whereas Cleopatra mandarin provided a higher-quality prey/feeding substrate for *E. stipulatus*, which may be related to its phytophagy, no differences were observed for the two specialists. As higher constitutive and faster inducible defense against *T. urticae* in sour orange relative to Cleopatra mandarin plants result in sour orange supporting lower *T. urticae* densities and plant damage, our results demonstrate that pest regulation by specialist natural enemies may be more effective when prey feed on better defended plants.

## Introduction

Phytoseiid mites (Mesostigmata; Phytoseiidae) have a diversity of lifestyles related to food utilization (McMurtry et al. 2013) ranging from specialized predators of herbivorous spider mites belonging to the genus *Tetranychus* (Prostigmata: Tetranychidae) (i.e., *Phytoseiulus persimilis* Athias-Henriot) to omnivorous pollen feeders that also feed on microarthropods and on plant cell-sap (i.e., the genus *Euseius*). Intermediate diet specializations are common in this family (McMurtry et al. 2013). In Spanish citrus orchards, the herbivore *Tetranychus urticae* Koch, a key pest of clementine mandarins (Pascual Ruiz et al. 2014), and the phytoseiids *P. persimilis*, *Euseius stipulatus* (Athias-Henriot) and *Neoseiulus californicus* (McGregor) coexist. These predators play a key role in the biological control of *T. urticae* in this agroecosystem (Aguilar-Fenollosa et al. 2011a, b; Pascual Ruiz et al. 2014; Pérez-Sayas et al. 2015). Whereas *P. persimilis* is a strict entomophagous species, *E. stipulatus* is a generalist omnivore suspected to feed on plant cell-sap. *Neoseiulus californicus* can also feed on tetranychid mites and plant-derived food (i.e., pollen) but most probably does not engage in plant cell-sap feeding (Adar et al. 2012; McMurtry and Croft 1997; McMurtry et al. 2013).

Cruz-Miralles et al. (2019) showed that *E. stipulatus* can induce plant defense responses in citrus similar to zoophytophagous predators (Dumont et al. 2018). These authors focused their study on two citrus species: sour orange, *Citrus aurantium* L., and Cleopatra mandarin, *Citrus reshni* hort. ex Tan., as they display extreme resistance and susceptibility to *T. urticae*, respectively (Agut et al. 2014; Bruessow et al. 2010). Sour orange is cultivated worldwide. Its high adaptability to all soil types, which induces good bearing and excellent fruit quality, made sour orange one of the most widely used rootstocks in the citrus industry in the Mediterranean area and in the Americas until the 1950s (Moreno et al. 2008). The emergence of the citrus quick decline disease, caused by the *Citrus tristeza virus* (CTV, Closteroviridae), killed almost 100 million citrus trees grafted on this rootstock worldwide (Moreno et al. 2008). This forced the massive replacement of sour orange by CTV-tolerant rootstocks, such as Cleopatra mandarin. These tolerant rootstocks, though, are more susceptible to *T. urticae* than sour orange (Bruessow et al. 2010). Indeed, this massive replacement is considered one of the triggers for the increasing prevalence of *T. urticae* as a pest of citrus (Bruessow et al. 2010). The differences in susceptibility to *T. urticae* between sour orange and Cleopatra mandarin are attributed to a higher constitutive and earlier inducible direct defense related to the oxylipin defensive pathway upon mite attack in sour orange compared to Cleopatra mandarin (Agut et al. 2014, 2015, 2016). These induced genotype-dependent responses closely match those triggered by *E. stipulatus* (Cruz-Miralles et al. 2019). Together with results by Gómez-Martínez et al. (2020) showing that this phytoseiid is able to obtain liquids by piercing a parafilm membrane, these results could be taken as an indirect evidence of the plant cell-sap feeding of *E. stipulatus*. Indeed, the cheliceral morphology typical of phytoseiid plant cell-sap feeders has been observed in different species of the genus *Euseius* (Adar et al. 2012). However, attempts to demonstrate feeding of *E. stipulatus* on leaves of lemon [*Citrus limon* (L) Burm. f.] and avocado (*Persea americana* Mill.) failed (Porres et al. 1975). This could be attributed to the plant feeding being cultivar-specific (Adar et al. 2012). Definite evidence for plant feeding is therefore needed to relate the observed plant defense responses to presumed herbivory by *E. stipulatus* (Cruz-Miralles et al. 2019).

Contrary to *E. stipulatus*, *N. californicus* is suspected not to feed on plant cell sap based on its cheliceral traits (Adar et al. 2012; McMurtry et al. 2013). *Phytoseiulus persimilis*, the third phytoseiid in the system, does not feed on plants (Magalhães and Bakker 2002; McMurtry and Croft 1997; McMurtry et al. 2013; Nomikou et al. 2003). These diet specializations could explain the responses of these phytoseiids when offered a choice between *T. urticae*-infested sour orange and Cleopatra mandarin plants Cabedo-López et al. (2019). Although a preference for less defended plants (i.e., infested Cleopatra mandarin rather than infested sour orange) was anticipated (keep in mind that prey densities should be higher on these plants), only *E. stipulatus* behaved as expected, whereas the other two phytoseiids showed no preference (Cabedo-López et al. (2019). This could be explained by the presumed herbivory of *E. stipulatus*, which would make this phytoseiid benefit from feeding directly on the less defended plant (Cleopatra mandarin) in periods when prey are scarce. This would not apply to the other two phytoseiids. Moreover, as there is evidence that fecundity of *P. persimilis* can double depending on the plant on which its prey, *T. urticae*, are feeding (Popov and Khudyakova 1989), we wondered whether prey profitability on sour orange and Cleopatra mandarin would be the same for these predators.

To challenge the hypotheses that (1) *E. stipulatus* is a zoophytophagous mite engaging in direct plant feeding and that (2) *T. urticae* profitability for the three phytoseiids considered is independent of the prey feeding substrate (sour orange and Cleopatra mandarin), we performed two experiments. In the first, we characterized leaf cuticular damage on sour orange and Cleopatra mandarin plants after exposure to *T. urticae* (positive control) and to each of the three phytoseiid species. In the second experiment, we evaluated prey and phytoseiid population growth (as a proxy of plant and prey profitability) on *T. urticae*-infested sour orange and Cleopatra mandarin plants. These results should help us to better understand direct and prey-mediated effects of plants on predators, and could provide clues for a more sustainable management of *T. urticae*.

## Materials and methods

Mite rearing and all experiments were carried out at controlled environmental conditions of 22 ± 5 °C; 60 ± 10% RH and 16:8 h L:D photoperiod with an illuminance of 5000 lx.

### Plant material

Three-month-old plants of sour orange and Cleopatra mandarin (with 10–12 true fully developed leaves) were used in our assays. They were obtained from seeds collected in trees cultivated at UJI campus and grown on vermiculite and peat (1:3; vol:vol) in 320-ml pots in a climatic chamber. To prevent any host-related maternal effect that could differentially affect the fitness of the mites used in our assays (Marshall and Uller 2007), mite stock colonies were maintained on either pesticide-free lemons [*Citrus limon* (L.) Burm f.], or bean plants (*Phaseolus vulgaris* L. cv. Buenos Aires Roja). These were also produced at UJI campus. *Typha* sp. pollen collected nearby was used to maintain phytoseiid stock colonies.

### Spider mite stock colony

The colony of *T. urticae* used in our assays, was initiated with specimens originally collected in 2001 in clementine orchards near UJI campus. Spider mites were maintained on lemons in a climatic chamber following Cruz-Miralles et al. (2019). In short, 8–10 lemons were set on top of a wooden structure placed in an open plastic box (40 × 30 × 8 cm) half-filled with water. The wooden structure maintained the lemons above the water, which prevented mites escaping from the rearing. Lemons were replaced weekly as needed.

### Phytoseiid stock colonies

The colonies of *E. stipulatus* and *P. persimilis* were started with specimens originally collected in 2012 in clementine orchards near UJI campus. Since then, colonies of these species have been maintained on rearing units using standard protocols (Pina et al. 2012). Basically, these consist of single bean leaflets placed upside down on a water-saturated sponge in a plastic tray (35 × 20 × 7 cm) with water. Strips of wet tissue were placed on the leaflet along its periphery to ensure a constant water supply to the phytoseiid and to prevent escape and contamination with other mite species. Twice a week, *E. stipulatus* and *P. persimilis* received pollen of *Typha* sp. and a mix of various stages of *T. urticae* as food, respectively. *Neoseiulus californicus* was regularly obtained from Koppert Biological Systems (SPICAL®) and a small colony was established on bean leaflets and supplied twice a week with both pollen and prey following the same procedure as before. For *E. stipulatus* and *N. californicus*, a small piece of black plastic sheet (2 cm^2^), folded in the shape of a roof, was placed in the rearing unit to provide a shelter and an oviposition substrate.

### Plant-feeding assays

To detect plant feeding in the three phytoseiids we used a technique based on Toluidine blue (TB) staining (Tanaka et al. 2004). This method allows rapid and inexpensive examination of defective cuticle over the entire leaf surface. Injured leaves exhibit intense blue stains due to rapid penetration of the dye into the leaf, whereas the cuticle of healthy, unwounded leaves resists the movement of TB into the leaf. Three replicates of one plant of both citrus genotypes per mite species (either a phytoseiid or *T. urticae*, which was used as a positive control) and the corresponding uninfested negative controls were performed. Three leaves per plant were selected and isolated from the rest of the plant by applying a ring of Tanglefoot® around their petioles. In the case of phytoseiids, these leaves received additional food (*Typha* sp. pollen for *E. stipulatus*, frozen *T. urticae* for *P. persimilis* and a mixture of both for *N. californicus*) to arrest phytoseiids on the leaves during the assay. Then, 30 adult females obtained from the stock colonies were transferred to those leaves (i.e., 10 mites per leaf) using a soft-bristle paintbrush. Infested plants were maintained in different climatic chambers where the additional food was supplied twice per week for 20 days. Subsequently, the three initially infested leaves per plant and three additional non-infested leaves from control plants were detached and directly immersed upside down in a 0.05% water (wt:wt) solution of toluidine for 5 h. Then, leaves were rinsed with distilled water using a sponge to eliminate any superficial residue of the dye on the leaves before observation under white light using a binocular microscope. Blue-stained areas corresponding to wound tissue (Tanaka et al. 2004) were attributed to mite feeding injury.

### *Tetranychus urticae* and phytoseiid population growth

The population growth of *T. urticae* and phytoseiids on sour orange and Cleopatra mandarin plants was evaluated. Three replicates of eight plants each per citrus genotype and phytoseiid species were considered. These replicates were run at different time periods, in summer for *N. californicus* and *P. persimilis* and autumn for *E. stipulatus*. The eight plants were infested with 25 adult females of *T. urticae*. These plants were maintained in climatic chambers separated by plant genotype during 8 days. Subsequently, three plants per genotype were cut in pieces and individually placed in a beaker with 500 ml of 70% (vol) ethanol. This mixture was stirred for 10 min with a glass stirring rod. Then, the suspension was poured onto a cellulose nitrate filter with a pore size of 0.45 µm (Sartorius Stedim Biotech, Barcelona, Spain) fitted to a filtration unit PSF 500/500 ml (Thermo Fisher Scientific, Sant Cugat del Vallès, Spain). Spider mites (all stages) retained on the filter were counted under a binocular microscope. Mean spider mite density and population composition (i.e., % eggs, immature motile stages, and adults) per citrus genotype were calculated. The remaining five *T. urticae*-infested plants per genotype received three young (ca. 3-day-old) adult phytoseiid females obtained from the stock colonies. They were left undisturbed for five additional days. Then, they were processed as before and all stages of *T. urticae* and phytoseiids were counted. Based on these counts, mite population growth was assessed.

For each replicate, we estimated the instantaneous rate of increase (r_i_), as defined by Hall (1964) and Walthall and Stark (1997), of *T. urticae* before (day 0 to 8) and after (day 8 to 13) the introduction of the predator, as well as that of the phytoseiids (day 8 to 13). This rate, which measures population changes after a short period of observation, was calculated according to the equation r_i_ = ln (N_f_/N_o_)/t, where N_f_ and N_o_ are the final and initial number of total individuals (i.e., all stages combined), and t refers to the number of days the experiment was run.

Mean mite densities and r_i_ on each host were compared using ANOVA. In the case of the r_i_ of *T. urticae*, the factors plant genotype, replicate, and their interaction were included in the analysis. For the r_i_ of phytoseiids, additionally *T. urticae* density and its 2- and 3-way interactions with genotype and replicate were considered. The software R v.3.5.3 was used (R Core Team 2020). To check whether the composition of mite populations (% eggs, immature motile stages and adults of either *T. urticae* or phytoseiids) was affected by the host plant/prey feeding substrate, we used contingency tables and the χ^2^ test. IBM SPSS Statistics 23 was used.

## Results

### Wound tissue associated to direct plant feeding occurs in *Euseius stipulatus* and *Tetranychus urticae*

**Fig. 1:**
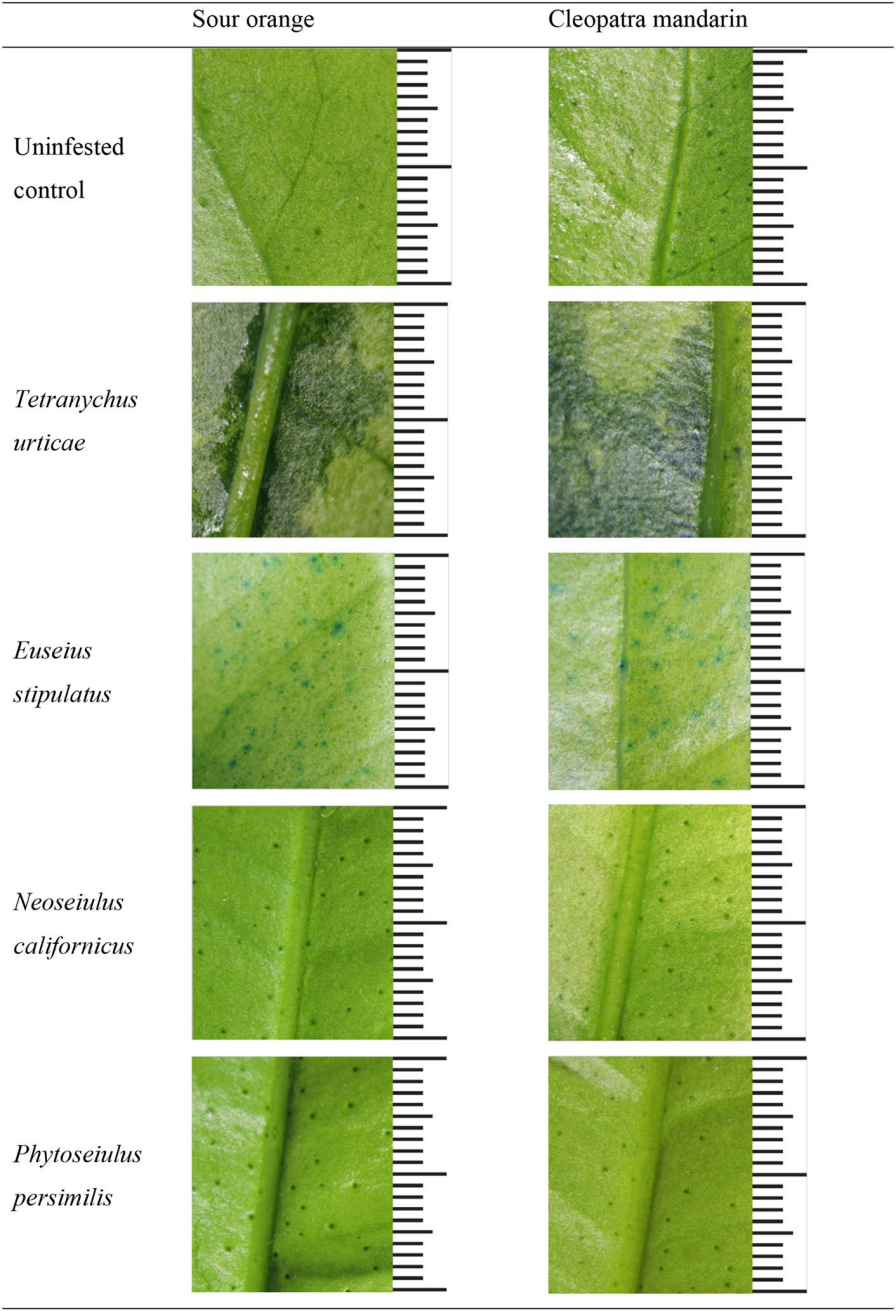
Toluidine blue staining patterns of the abaxial surfaces of leaves of sour orange and Cleopatra mandarin. Infested plants received 30 adult females of the corresponding mite species and were processed 20 days later (scale: 2 cm; each bar represents 1 mm)

Evidence of cuticle damage on the abaxial surfaces of leaves of sour orange and Cleopatra mandarin exposed to either *T. urticae* or *E. stipulatus* could be easily identified with TB staining (Fig. 1). No sign of cuticular injury was observed for the other two phytoseiids although specimens of both species could be observed on the plants at the end of the assay. Damage caused by *T. urticae* corresponded to large blue-colored patches matching the areas formerly occupied by spider mite colonies. Injury caused by *E. stipulatus* was less conspicuous and consisted of much smaller areas (dots) regularly covering the leaf surface of both citrus genotypes, most probably corresponding to individual feeding punctures as *E. stipulatus* do not live in colonies. Colored areas were frequently observed in the vicinity of stomata.

### Effect of plant host on prey and predator population growth

As expected, mean densities of *T. urticae* before the release of phytoseiids were higher on Cleopatra mandarin than on sour orange (grand mean ± SE = 205.23 ± 15.02 and 149.12 ± 11.93 specimens per plant, respectively), with higher values observed in the assays carried out in summer (i.e., *N. californicus* and *P. persimilis*) than in autumn (i.e., *E. stipulatus*) (Tables 1, 2 and 3). These densities corresponded to higher instantaneous rates of increase of *T. urticae* in Cleopatra mandarin compared to sour orange (Tables 1, 2 and 3), with the same seasonal differences as above. The introduction of the phytoseiids reduced these rates, which became negative in all cases (grand mean ± SE for *E. stipulatus* = –0.007 ± 0.032) and independent of the plant genotype, except for *N. californicus*. For this phytoseiid, higher reductions of *T. urticae* densities were observed on sour orange. As a consequence, *T. urticae* densities at the end of the assay were lower than 5 days earlier, except in the presence of *E. stipulatus*. This generalist predator was the only phytoseiid whose instantaneous rate of increase depended on the plant genotype (Table 1). It was negative on sour orange and positive but around one order of magnitude lower than those of the other two phytoseiids on Cleopatra mandarin. In contrast, the instantaneous rate of increase of *N. californicus* and *P. persimilis* did not depend on the plant genotype. No relationship between r_i_ and prey density was observed for any of the phytoseiids (Table 3). The plant genotype*replicate was the only interaction that was significant in four cases (Tables 1, 2 and 3). In these four cases, sour orange in one of the three replicates had lower values than most of the other plant genotype*replicate values (see Tables 1 and 3). This situation never affected the species-specific relative suitability of the two plant genotypes considered.

**Table 1:** Mean (± SE) density (number of mites per plant) and instantaneous rate of increase, r_i_ (day^−1^) of *Tetranychus urticae* before (day 8) and after (day 13) the introduction of *Euseius stipulatus*, as well as those of the phytoseiid (day 13)

Plant genotype	*T. urticae*				*E. stipulatus*	
	Density day 8	r_i_ (no phytoseiid)	Density day 13	r_i_ (phytoseiid)	Density day 13	r_i_
SO	74.4 ± 9.0	0.152 ± 0.025	120.3 ± 16.3	− 0.008 ± 0.133	2.3 ± 0.3	− 0.053 ± 0.031
CM	112.2 ± 7.7	0.186 ± 0.006	118.1 ± 41.2	0.017 ± 0.051	4.3 ± 0.9	0.037 ± 0.050
A*B*C	–	–	–	–	F_5,24_ = 2.28, P = 0.08	F_5,22_ = 1.87, P = 0.14
A*B	F_5,12_ = 5.46, P = 0.02	F_5,12_ = 5.46, P = 0.02	F_5,24_ = 3.13, P = 0.06	F_5,24_ = 4.47, P = 0.02	F_5,24_ = 0.34, P = 0.57	F_5,24_ = 0.47, P = 0.63
	SO_R3_ ≤ SO_R2_ = SO_R1_ < CM_R1_ = CM_R2_ = CM_R3_	SO_R3_ ≤ SO_R2_ = SO_R1_ < CM_R1_ = CM_R2_ = CM_R3_		SO_R1_ = SO_R3_ = CM_R1_ = CM_R3_ = CM_R2_ ≤ SO_R2_		
A*C	–	–	–	–	F_2,27_ = 1.12, P = 0.30	F_2,27_ = 2.16, P = 0.15
B*C	–	–	–	–	F_3,26_ = 0.19, P = 0.83	F_3,26_ = 0.16, P = 0.86
A	–	–	F_1,28_ = 0.02, P = 0.88	–	F_1,28_ = 7.28, P = 0.01	F_1,28_ = 4.29, P = 0.049
			SO = CM		SO < CM	SO < CM
B	–	–	F_2,27_ = 9.27, P < 0.001	–	F_2,27_ = 1.71, P = 0.20	F_2,27_ = 2.14, P = 0.14
			R1 = R3 > R2		R1 = R3 = R2	R1 = R3 = R2
C	–	–	–	–	F_1,28_ = 0.67, P = 0.42	F_1,28_ = 2.02, P = 0.17

Densites and r_i_ values in sour orange (SO) and Cleopatra mandarin (CM) were compared using ANOVA with plant genotype (A) and replicate (B) as factors in the case of *T. urticae*. For the phytoseiid, *T. urticae* density at day 8 (C) was also added to the analysis. All possible interactions were initially taken into account but eventually discarded when non-significant (*P* > 0.05). Mean values correspond to the average of three replicates (R1, R2, R3) of either three (for *T. urticae* densities and r_i_ at day 8) or five (for *T. urticae* and *E. stipulatus* densities and r_i_ at day 13) plants per plant genotype

**Table 2:** Mean (± SE) density (number of mites per plant) and instantaneous rate of increase, r_i_ (day^−1^) of *Tetranychus urticae* before (day 8) and after (day 13) the introduction of *Neoseiulus californicus*, as well as those of the phytoseiid (day 13)

Plant genotype	*T. urticae*				*N. californicus*	
	Density day 8	r_i_ (no phytoseiid)	Density day 13	r_i_ (phytoseiid)	Density day 13	r_i_
SO	169.1 ± 18.1	0.211 ± 0.024	128.0 ± 7.5	− 0.147 ± 0.014	6.7 ± 1.0	0.145 ± 0.031
CM	233.6 ± 20.4	0.278 ± 0.011	188.4 ± 9.3	− 0.073 ± 0.003	6.4 ± 1.5	0.116 ± 0.065
A*B*C	–	–	–	–	F_5,24_ = 1.45, P = 0.24	F_5,24_ = 2.01, P = 0.11
A*B	F_5,12_ = 0.30, P = 0.75	F_5,12_ = 0.38, P = 0.69	F_5,24_ = 0.08, P = 0.92	F_5,24_ = 0.27, P = 0.76	F_5,24_ = 1.06, P = 0.36	F_5,24_ = 1.96, P = 0.16
A*C	–	–	–	–	F_2,27_ = 0.38, P = 0.54	F_2,27_ = 0.10, P = 0.75
B*C	–	–	–	–	F_3,26_ = 1.10, P = 0.35	F_3,26_ = 2.10, P = 0.14
A	F_1,16_ = 52.71, P < 0.001	F_1,16_ = 62.73, P < 0.001	F_1,28_ = 9.45, P < 0.01	F_1,28_ = 9.02, P < 0.01	F_1,28_ = 0.08, P = 0.78	F_1,28_ = 0.48, P = 0.49
	SO < CM	SO < CM	SO < CM	SO < CM	SO = CM	SO = CM
B	F_2,15_ = 12.25, P < 0.001	F_2,15_ = 15.74, P < 0.001	F_2,27_ = 0.21, P = 0.81	F_2,27_ = 0.15, P = 0.86	F_2,27_ = 2.58, P = 0.09	F_2,27_ = 2.62, P = 0.09
	R1 = R3 > R2	R1 = R3 > R2	R1 = R2 = R3	R1 = R2 = R3	R1 = R2 = R3	R1 = R3 = R2
C	–	–	–	–	F_1,28_ = 1.12; P = 0.30	F_1,28_ = 1.82, P = 0.19

Densites and r_i_ values in sour orange (SO) and Cleopatra mandarin (CM) were compared using ANOVA with plant genotype (A) and replicate (B) as factors in the case of *T. urticae*. For the phytoseiid, *T. urticae* density at day 8 (C) was also added to the analysis. All possible interactions were initially taken into account but eventually discarded when non-significant (*P* > 0.05). Mean values correspond to the average of three replicates (R1, R2, R3) of either three (for *T. urticae* densities and r_i_ at day 8) or five (for *T. urticae* and *N. californicus* densities and r_i_ at day 13) plants per plant genotype

**Table 3:** Mean (± SE) density (number of mites per plant) and instantaneous rate of increase, r_i_ (day^−1^) of *Tetranychus urticae* before (day 8) and after (day 13) the introduction of *Phytoseiulus persimilis*, as well as those of the phytoseiid (day 13)

Plant genotype	*T. urticae*				*P. persimilis*	
	Density day 8	r_i_ (no phytoseiid)	Density day 13	r_i_ (phytoseiid)	Density day 13	r_i_
SO	195.7 ± 43.5;	0.257 ± 0.031	76.9 ± 27.6	− 0.315 ± 0.073	13.4 ± 3.5	0.271 ± 0.063
CM	272.1 ± 28.9	0.297 ± 0.014	76.0 ± 13.7	− 0.317 ± 0.069	15.4 ± 3.2	0.304 ± 0.048
A*B*C	–	–	–	–	F_5,24_ = 0.23, P = 0.63	F_5,24_ = 0.08, P = 0.78
A*B	F_5,12_ = 1.37, P = 0.30	F_5,10_ = 5.22, P = 0.03	F_5,10_ = 0.93, P = 0.41	F_5,24_ = 2.80, P = 0.08	F_5,24_ = 0.15, P = 0.86	F_5,24_ = 0.08, P = 0.78
		SO_R3_ < SO_R1_ = SO_R2_ < CM_R1_ = CM_R2_ = CM_R3_				
A*C	–	–		–	F_2,27_ = 0.03, P = 0.86	F_2,27_ = 0.01, P = 0.95
B*C	–	–		–	F_3,26_ = 0.12, P = 0.89	F_3,26_ = 0.07, P = 0.93
A	F_1,16_ = 64.25, P < 0.001	F_1,16_ = 71.89, P < 0.001	F_1,28_ = 0.01, P = 0.96	F_1,28_ = 0.01, P = 0.97	F_1,28_ = 0.66, P = 0.42	F_1,28_ = 0.62, P = 0.44
	SO < CM	SO < CM	SO = CM	SO = CM	SO = CM	SO = CM
B	F_2,15_ = 30.41, P < 0.001	F_2,15_ = 39.22, P < 0.001	F_2,27_ = 2.59, P = 0.09	F_2,27_ = 3.33, P = 0.05	F_2,27_ = 6.50, P < 0.01	F_2,27_ = 6.55, P < 0.01
	R1 = R2 > R3	R1 = R2 > R3	R1 = R2 = R3	R1 = R2 = R3	R1 = R2 > R3	R1 = R2 > R3
C	–	–	–	–	F_1,28_ = 0.72, P = 0.80	F_1,28_ = 0.09, P = 0.78

Densites and r_i_ values in sour orange (SO) and Cleopatra mandarin (CM) were compared using ANOVA with plant genotype (A) and replicate (B) as factors in the case of *T. urticae*. For the phytoseiid, *T. urticae* density at day 8 (C) was also added to the analysis. All possible interactions were initially taken into account but eventually discarded when non-significant (*P* > 0.05). Mean values correspond to the average of three replicates (R1, R2, R3) of either three (for *T. urticae* densities and r_i_ at day 8) or five (for *T. urticae* and *P. persimilis* densities and r_i_ at day 13) plants per plant genotype

The composition of the population of *T. urticae* 8 days after infestation, just before the introduction of the phytoseiids, showed that immature motiles were the most abundant stage in sour orange (55.7%), followed by eggs (35.5%) and adults (8.9%), whereas eggs (49.2%) predominated in Cleopatra mandarin, followed by immature motiles (42.5%) and adults (8.3%) (Table 4). These plant genotype-specific distributions were different except for plants exposed to *N. californicus* (Table 4) and changed after the introduction of the phytoseiids (Table 4). At the end of the assay, immature motiles were the most abundant stage (75.8%) followed by eggs (13.5%) and adults (10.7%) irrespective of the plant genotype. This decreasing egg abundance could be taken as indicative of this stage suffering higher predation rates than the other two but also of reduced oviposition. Phytoseiid population compositions did not change with the host plant and included immature stages, showing that the three phytoseiid species could reproduce on both host plants during the experiment (Tables 1, 2 and 3).

**Table 4:** Composition (% eggs, immature motile stages, and adults) of the populations of *Tetranychus urticae* 8 and 13 days after infestation with 25 females per plant and that of phyoseiids on day 13 (i.e., 5 days after the release of three females per plant) in sour orange (SO) and Cleopatra mandarin (CM)

Phytoseiid species	Plant genotype	*T. urticae* population composition				Phytoseiid population composition	
		Day 8	χ^2^ (df = 1), P	Day 13	χ^2^ (df = 1), P	Day 13	χ^2^ (df = 1), P
*Euseius stipulatus*	SO		10.219, <0.01		0.882, 0.64		0.055, 0.97
	CM						
*Neoseiulus californicus*	SO		3.175, 0.21		2.227, 0.33		0.149, 0.93
	CM						
*Phytoseiulus persimilis*	SO		30.410, < 0.001		1.775, 0.41		0.115, 0.94
	CM						

■ Eggs, □ immature motile stages,  adults

Means of three replicates of either three (*T. urticae* populations at day 8) or five (*T. urticae* and *E. stipulatus* populations day 13) plants per plant genotype. Comparisons were performed using contingency tables and χ^2^ tests

## Discussion

As expected, we found that *T. urticae* can develop faster on Cleopatra mandarin than on sour orange (Agut et al. 2014, 2016). The seasonal differences observed in our study have been repeatedly reported for this mite (Aucejo-Romero et al. 2004; Urbaneja-Bernat et al. 2019). Moreover, our results also provide evidence that *E. stipulatus* can directly feed on plants. Therefore, this phytoseiid should be considered as a true zoophytophagous predator (Dumont et al. 2018). Contrary to the other two phytoseiids included in this study, this herbivory would explain why *E. stipulatus* grew faster on less defended Cleopatra mandarin than on better defended sour orange plants, as shown by the instantaneous rates of increase recorded.

Lemon is a cultivated plant on which *E. stipulatus* is commonly encountered in its native Mediterranean region (Jaques et al. 2015; Vela et al. 2017). However, Porres et al. (1975) could not demonstrate feeding on lemon leaves labeled with radioactive phosphoric acid. Adar et al. (2012) suspected that phytoseiid plant feeding could be cultivar specific. In agreement with our first hypothesis, our results prove that *E. stipulatus* can feed on at least the two citrus species tested here. Whether it is also capable of feeding on other plants on which this phytoseiid occurs (Aguilar-Fenollosa et al. 2011b; Aucejo et al. 2003; Ferragut and Escudero 1997) deserves further research. It remains unclear whether *E. stipulatus* only obtains water or also nutrients from the plant, as in other plant-feeding phytoseiids (Adar et al. 2012). Plant feeding on pepper leaves by the closely related species *Euseius scutalis* (Athias-Henriot) left discrete holes in the leaf surface surrounded by intact cells (Adar et al. 2015), which is coherent with the dot-like feeding wounds observed with TB staining for *E. stipulatus*. Our results point at phytophagy as the most likely cause for the observed plant defense responses to *E. stipulatus* (Cruz-Miralles et al. 2019). However, other triggers (i.e., oviposition, excretion, or walking) (Hilker and Fatouros 2015; Hilker and Meiners 2010; Howe and Jander 2008; Karban 2020; Schuman and Baldwin 2016; Wu and Baldwin 2010) cannot be discarded. Herbivory could also, at least partly, explain the negative r_i_ observed in sour orange, which would make additional food sources indispensable for survival of *E. stipulatus* on this host plant.

Our second hypothesis was that the profitability of *T. urticae* as prey would be predator-specific and independent of the prey feeding substrate. This hypothesis proved partially correct. On the one hand, the instantaneous rates of increase of the three phytoseiids were species specific. On the other hand, they were independent of the host plant for *N. californicus* and *P. persimilis* but depended on it for *E. stipulatus*.

The omnivore *E. stipulatus* is considered to perform poorly on well-established *T. urticae* colonies because of its inability to enter and move within the dense webs produced by *T. urticae* (Sabelis and Bakker 1992). This may explain why the abundant prey present may have not been easily accessible for this phytoseiid in our experiments. This may have triggered this phytoseiid to feed on the host plant. Such a shift would not be possible for the other two phytoseiids, as shown by our results. Indeed, *P. persimilis* cannot be maintained on citrus plants for more than 24 h without prey (Cruz-Miralles et al. 2021). Therefore, *E. stipulatus* would benefit from feeding on relatively less defended Cleopatra mandarin rather than better protected sour orange. As a consequence, *E. stipulatus* r_i_ was negative in the three replicates involving sour orange and in one of those involving Cleopatra mandarin (data not shown). Our results support the hypotheses that *E. stipulatus* poses a relatively low predation risk to *T. urticae* and that it has a marked preference for other food resources. The latter was confirmed by molecular gut-content analyses of field-collected *E. stipulatus* specimens (Pérez-Sayas et al. 2015). That study showed that *E. stipulatus* prefers to feed on alternative food, including other phytoseiids, even when tetranychid prey was abundant. The same adaptations of *T. urticae* that make Cleopatra mandarin a better host plant may occur in *E. stipulatus*. This would explain why Cleopatra mandarin was more favorable for this phytoseiid, both directly (plant feeding) and indirectly (via the prey), than sour orange.

In contrast, the instantaneous rate of increase of the other two phytoseiid species did not change with the host plant, which can be taken as evidence that prey profitability does not depend on the prey’s host plant. Both predators proved much more efficient at controlling *T. urticae* than *E. stipulatus*. This is in agreement with previous studies (Abad-Moyano et al. 2009; Escudero and Ferragut 2005; Ferragut and Garcia-Mari 1987), and highlights the importance of maintaining these two specialist species in citrus orchards for satisfactory control of *T. urticae* (Aguilar-Fenollosa et al. 2011a, b).

To conclude, our results provide evidence that *E. stipulatus* is a zoophytophagous mite, which can engage in direct plant feeding in sour orange and Cleopatra mandarin, whereas neither *N. californicus* nor *P. persimilis* did so. Although the profitability of *T. urticae* as prey for *N. californicus* and *P. persimilis* seems to be the same on sour orange and Cleopatra mandarin, the latter was a better host plant for *E. stipulatus*. As higher constitutive and faster inducible defenses against *T. urticae* in sour orange relative to Cleopatra mandarin plants result in the former supporting lower *T. urticae* densities and plant damage (Bruessow et al. 2010; Agut et al. 2015), our results show that pest regulation by specialist natural enemies may be more effective when prey feeds on better defended plants (i.e., sour orange rather than Cleopatra mandarin). These results demonstrate that direct and indirect defense could work synergistically in agricultural important crops such as citrus. Sour orange can no longer be used as a rootstock because CTV is nowadays present in almost all citrus growing areas worldwide (EPPO 2020). However, its use as a companion plant or in intercropping systems with other citrus species should be further evaluated as a means of promoting the conservation of *N. californicus* and *P. persimilis*.

Aguilar-Fenollosa et al. (2011b) observed a higher abundance of generalist pollen feeders (i.e., *E. stipulatus*) in citrus associated with a diverse wild cover crop relative to those associated with a sown cover of the Poaceae *Festuca arundinacea* Schreb. This higher abundance corresponded to reduced control of *T. urticae*. This was caused by the specialist phytoseiids (i.e., *N. californicus* and *P. persimilis*) suffering increased competition from the generalist pollen feeders. Eventually, this could result in the specialists being virtually wiped out from the system. Indeed, the molecular gut-content analyses carried out by Pérez-Sayas et al. (2015) revealed the occurrence of intraguild (IG) predation in field-collected specimens of *E. stipulatus*, which were positive for either *N. californcius*, *P. persimilis,* or both. This result is coherent with those of Abad-Moyano et al. (2010), who identified *E. stipulatus* as the IG-predator in this system. Therefore, the implementation of cultural practices favoring *N. californicus* and *P. persimilis* but not *E. stipulatus* (i.e., the use of sour orange) could enhance the natural regulation of *T. urticae* in citrus. Moreover, because the volatile blend released from sour orange plants following *T. urticae* infestation induces resistance in Cleopatra mandarin against this mite but not vice-versa (Agut et al. 2016; Cabedo-López et al. 2019), this effect could be also exploited in mixed cropping systems to increase the resilience of the crop.

## Data Availability

Raw data deposited at UJI Public Digital Repository.
